# *GmFLD*, a soybean homolog of the autonomous pathway gene *FLOWERING LOCUS D*, promotes flowering in *Arabidopsis thaliana*

**DOI:** 10.1186/s12870-014-0263-x

**Published:** 2014-10-07

**Authors:** Qin Hu, Ye Jin, Huazhong Shi, Wannian Yang

**Affiliations:** Hubei Key Laboratory of Genetic Regulation and Integrative Biology, School of Life Sciences, Central China Normal University, Wuhan, 430079 People’s Republic of China; Department of Chemistry and Biochemistry, Texas Tech University, Lubbock, TX 79409 USA

**Keywords:** Autonomous pathway, Deacetylation, Demethylation, *FLOWERING LOCUS D*, Flowering transition, *GmFLD*, Histone demethylase, Soybean

## Abstract

**Background:**

Flowering at an appropriate time is crucial for seed maturity and reproductive success in all flowering plants. Soybean (*Glycine max*) is a typical short day plant, and both photoperiod and autonomous pathway genes exist in soybean genome. However, little is known about the functions of soybean autonomous pathway genes. In this article, we examined the functions of a soybean homolog of the autonomous pathway gene *FLOWERING LOCUS D* (*FLD*), *GmFLD* in the flowering transition of *A. thaliana*.

**Results:**

In soybean, *GmFLD* is highly expressed in expanded cotyledons of seedlings, roots, and young pods. However, the expression levels are low in leaves and shoot apexes. Expression of *GmFLD* in *A. thaliana* (Col) resulted in early flowering of the transgenic plants, and rescued the late flowering phenotype of the *A. thaliana fld* mutant. In *GmFLD* transgenic plants (Col or *fld* background), the *FLC* (*FLOWERING LOCUS C*) transcript levels decreased whereas the floral integrators, *FT* and *SOC1*, were up-regulated when compared with the corresponding non-transgenic genotypes. Furthermore, chromatin immuno-precipitation analysis showed that in the transgenic rescued lines (*fld* background), the levels of both tri-methylation of histone H3 Lys-4 and acetylation of H4 decreased significantly around the transcriptional start site of *FLC*. This is consistent with the function of GmFLD as a histone demethylase.

**Conclusions:**

Our results suggest that GmFLD is a functional ortholog of the Arabidopsis FLD and may play an important role in the regulation of chromatin state in soybean. The present data provides the first evidence for the evolutionary conservation of the components in the autonomous pathway in soybean.

**Electronic supplementary material:**

The online version of this article (doi:10.1186/s12870-014-0263-x) contains supplementary material, which is available to authorized users.

## Background

Flowering at an appropriate time is crucial for seed maturity and reproductive success in all flowering plants. Multiple flowering promotion pathways that respond to both environmental cues and endogenous factors have been evolved in plants to properly regulate flowering time. In the model plant *A. thaliana*, the photoperiod and vernalization pathways monitor seasonal changes in day length and temperature. These two pathways are responsible for initiating flowering in response to long days or prolonged cold temperatures (vernalization). The autonomous pathway, together with the gibberellin acid (GA) pathway, integrates signals from the developmental state of the plant and promotes flowering constitutively [1-3].

In the photoperiod pathway, CONSTANS (CO) is the key protein and its expression is regulated by GI (GIGANTEA), which is under the control of the circadian clock [4]. During long days, CO protein accumulates to high enough levels to promote floral transition, and as a result, up-regulates the expression of *FT* (*FLOWERING LOCUS T*) to initiate flowering [4,5]. Both the vernalization pathway and autonomous pathway promote flowering through repression of the expression of *FLC* (*FLOWERING LOCUS C*), a central repressor of flowering in *A. thaliana* [6,7]. *FLC* is activated by *FRI* (*FRIGIDA*), a transcription factor with coiled coil motifs [8-10]. Natural *A. thaliana* winter annuals contain the dominant alleles of *FRI* and require vernalization, which represses *FLC* expression, for flowering [8,9,11]. Vernalization represses the expression of *FLC* by regulating the chromatin status of the *FLC* locus, and the underlying mechanism of this regulation was discussed in several critical reviews [2,12-15]. In contrast to winter annuals, natural rapid-cycling accessions do not have the functional *FRI. FLC* is suppressed constitutively by the so-called autonomous pathway genes, including *FCA* (*FLOWERING LOCUS CA*), *FY* (*FLOWERING LOCUS Y*), *FPA* (*FLOWERING LOCUS PA*), *FVE* (*FLOWERING LOCUS VE*), *LD* (*LUMINIDEPENDENS*), *FLD* (*FLOWERING LOCUS D*), and *FLK* (*FLOWERING LOCUS KH DOMAIN*) [16]. Among these autonomous pathway components, FCA, FPA, FY and FLK participate in the RNA regulatory process in controlling flowering [14,15,17], whereas LD, FVE, and FLD are involved in the regulation of the chromatin modification state [2,14,15].

*FLD* encodes a plant ortholog of the human Lys-Specific Demethylase 1 (LSD1) protein. FLD functions in histone H3K4 demethylation and H3/H4 deacetylation to repress the expression of *FLC* [18-20]. In vivo, FLD is a sumoylation target of SIZ1 [SAP (scaffold attachment factor, acinus, protein inhibitor of activated signal transducer and activator of transcription) and Miz1 (Msx2-interacting zinc finger), SIZ], an E3 ligase in Arabidopsis. SUMO conjugation to FLD inhibits its repression activity for *FLC* expression and is required for full activation of *FLC* in a *FRI* background [21]. Recently, Zhang et al. [22] reported that *FLD* expression is regulated by *BRZ1* (*BRASSINAZOLE-RESISTANT1*) in a CYP20-2 dependent manner. Hence FLD may mediate brassinosteroid-controlled flowering regulation in Arabidopsis. FLD physically interacts with HDA6 to act synergistically in controlling the flowering of *A. thaliana* [20]. In *A. thaliana* genome, two other *FLD* homolog, *LSD1-LIKE1* (*LDL1*) and *LSD1-LIKE2* (*LDL2*), act in partial redundancy with *FLD* to repress *FLC* expression. However, *LDL1* and *LDL2* act independently of *FLD* in the silencing of *FWA* (*FLOWERING WAGENINGEN*), a homeodomain-containing transcription factor. The *FWA* gene is silenced in the sporophyte and only expressed in the female gamete and extra-embryonic endosperm tissue in a maternal-imprinted manner [19].

Soybean is a typical short-day plant and the photoperiod sensitivity of different soybean cultivars is associated with their distribution range. Hence, soybean is also a short-day model plant for studying photoperiod response, and much progress has been made in identifying functions of the genes in the photoperiod pathway in soybean. To the best of our knowledge, at least ten *FT* homologs were experimentally identified in the soybean genome [23]. Among these FT homologs, GmFT2a and GmFT5a are thought to be the florigen in soybean, and their expressions are regulated by the *PHYA*-mediated photoperiodic regulation system [24] as well as the classical maturity locus *E1* encoding a novel plant transcription factor, which plays a pivotal role in controlling soybean flowering [25]. Ectopic expression of *GmFT2a* and *GmFT5a* in *A. thaliana* resulted in premature flowering [24] and *GmFT2a* over-expression in soybean resulted in precocious flowering independent of photoperiod [26]. *CO* has four homologs in soybean, *GmCOL1a*, *GmCOL1b*, *GmCOL2a* and *GmCOL2b*, and each of them can fully complement the late flowering effect of the *co* mutant in *A. thaliana* [27,28]. *GIGANTEA* has three soybean homologs, *GmGI1a*, *GmGI1* and *GmGI2*, whose responses to circadian clock and photoperiod are different from each other [27,29,30]. *GmGI1a* is the classical maturity locus *E2*, who has multiple functions involved in the circadian clock and flowering [27,29]. Hence, although soybean is a short-day plant, which is different from *A. thaliana*, the photoperiod pathway seems to be conserved between these two species [31].

Based upon the draft sequence of the soybean genome [32], homologs of autonomous pathway genes were also identified from the genome through bioinformatics analysis [27,31,33]. However, study on this group of genes has been limited. In this paper, the functions of the soybean *FLD* ortholog, GmFLD, were tested experimentally. Heterologous expression of *GmFLD* in *A. thaliana* resulted in early flowering of the transgenic plants and could partially complement the late flowering phenotype of *fld* mutants. In the *GmFLD* transgenic *A. thaliana* (Col or *fld* background), *FLC* transcript levels decreased and the floral integrator genes *FT* and *SOC1* increased significantly. In the complementing transgenic lines, both histone H3 lysine4 trimethylation (H3K4me3) and H4 acetylation decreased around the transcriptional start site of *FLC*. Our results suggest that GmFLD is a functional soybean ortholog of FLD and may play an important role in the regulation of the chromatin modification state in soybean.

## Results

### Soybean has four *FLD* homologs

**Figure 1:**
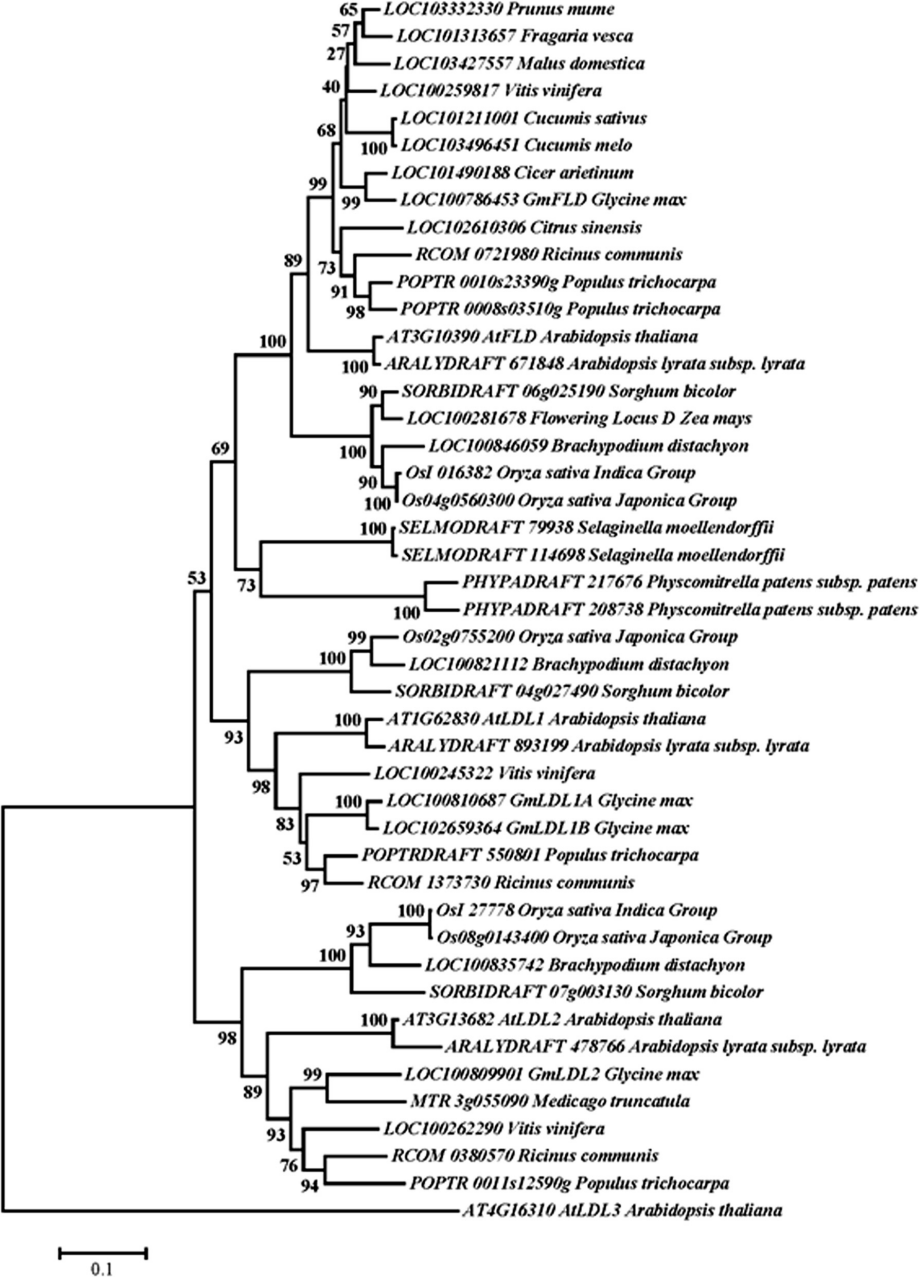
**Phylogenetic tree of FLD homologs from soybean and other plant species.** The phylograph was generated by the Neighbor-Joining method using Mega 5.0 [55]. Bootstrap analysis was performed in 1000 sampling replicates.

In *A. thaliana*, *FLD* has other two homologs: *LDL1* and *LDL2*. These homologs act redundantly with *FLD* to repress *FLC* transcription [19]. By searching the NCBI soybean genome database using the Arabidopsis FLD protein sequence, four FLD homologs (E value = 0.0) were found: LOC100786453 (Glyma02g18610), LOC100810687 (Glyma09g31770), LOC100783933 (Glyma07g09980), and LOC100809901 (Glyma06g38600) with identity of 73%, 57%, 53% and 52% respectively. The deduced amino acid sequences of these four genes were then blasted against the *A. thaliana* proteome database (TAIR10), and the results show that LOC100786453 (Glyma02g18610) has the highest homology to the Arabidopsis FLD (73% identity), LOC100783933 (Glyma07g09980) and LOC100810687 (Glyma09g31770) are more similar to LDL1 (65% and 71% identity respectively), and LOC100809901 (Glyma06g38600) is more related to LDL2 (66% identity). Phylogenetic analysis with FLD homologs from different plant species show that plant LSD1 homologs are divided into three subgroups: LOC100786453 (Glyma02g18610) is clustered with the Arabidopsis FLD, LOC100809901 (Glyma06g38600) is in the LDL2 cluster, and LOC100783933 (Glyma07g09980) and LOC100810687 (Glyma09g31770) belong to LDL1 cluster (Figure 1). Hence, LOC100786453 (Glyma02g18610) is designated as GmFLD, LOC100809901 (Glyma06g38600) is designated as GmLDL2, and LOC100783933 (Glyma07g09980) and LOC100810687 (Glyma09g31770) are designated as GmLDL1A and GmLDL1B, respectively.

**Figure 2:**
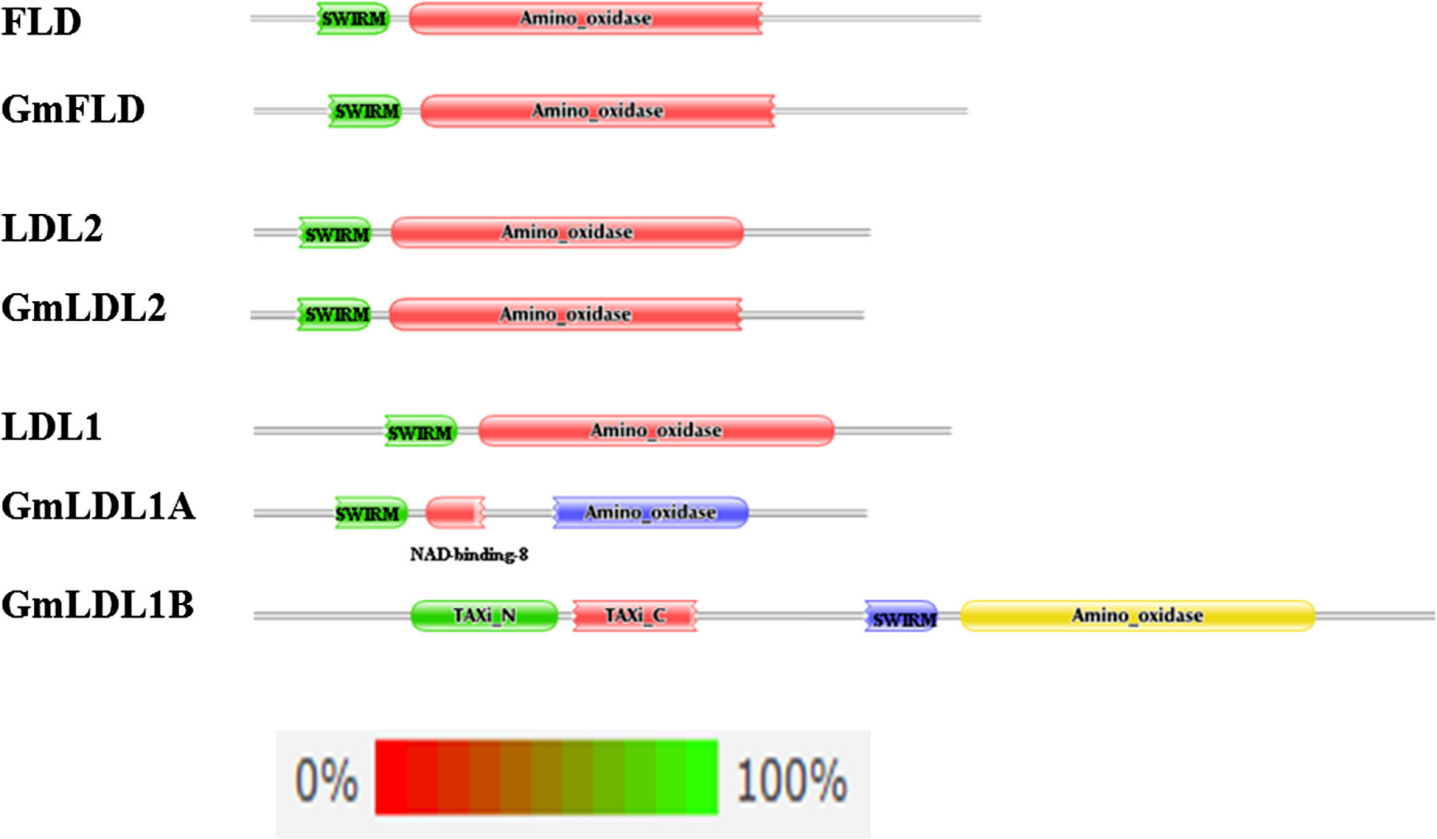
**Schematic domain structures of soybean FLD homologs.** The schemas were generated by online searching pfam27.0 database [56] with amino acid sequences of FLD and its homologs. Columns are colored according to the posterior probability.

Both GmFLD and GmLDL2 contain the SWIRM and Amine Oxidase domains (Figure 2) that are characteristic of the LSD1 group of histone demethylases [19]. The SWIRM and amino oxidase domains in GmFLD and GmLDL2 are organized in the same pattern as those in the Arabidopsis FLD and LDL2: SWIRM domain is at the N terminal while the C terminal contains the amine oxidase domain (Figure 2). However, in addition to the SWIRM and the amino oxidase domains, both GmLDL1A and GmLDL1B proteins contain new domains that are not present in the Arabidopsis: LDL1, LDL2 and FLD (Figure 2). GmLDL1A contains a NDA-binding-8 domain between the SWIRM domain and the amino oxidase domain, while GmLDL1B contains TAXi-N and TAXi-C domains at the N-terminal in front of the SWIRM domain. The NAD-binding-8 domain is involved in coenzyme binding [34], whereas the proteins containing TAXi domains are associated with proteolysis of phytopathogen xylanase secreted by the pathogen to degrade plant cell wall during plant pathogen infection [35]. In addition, both *GmLDL1A* and *GmLDL1B* differ from the Arabidopsis *LDL1* in that the soybean genes have intron (s) according to the annotations at NCBI and JGI databases (Additional file 1). Taken together, GmLDL1s probably diverged in functions from their counterpart LDL1 during evolution.

### *GmFLD* and *GmLDL2* exhibit different expression patterns from their Arabidopsis counterparts: *FLD* and *LDL2*

**Figure 3:**
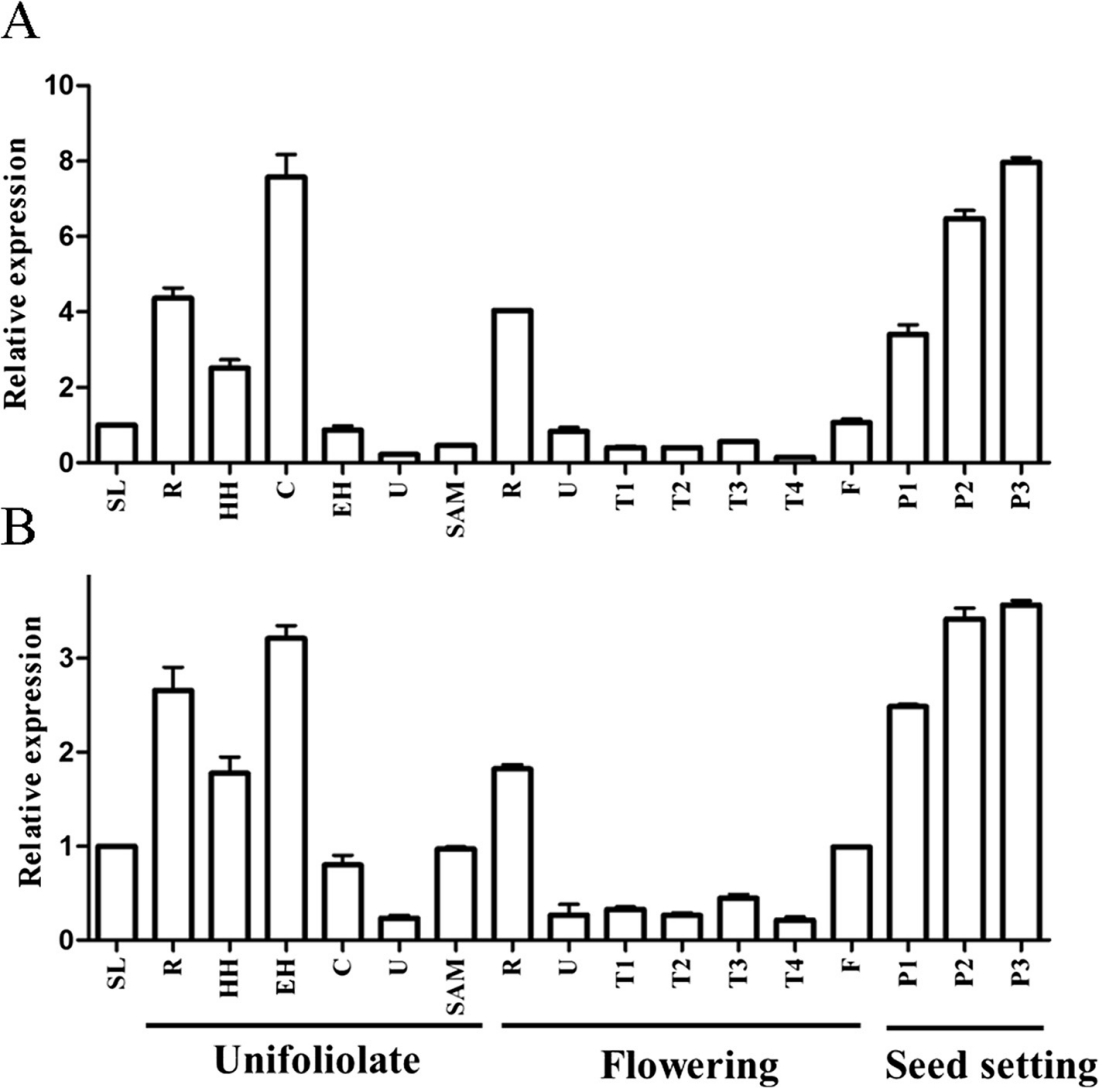
**The expression profiles of** ***GmFLD*** **(A) and** ***GmLDL2*** **(B).** SL, seedling; R, root; HH, hypocotyl; E, epicotyl; C, cotyledon; U, unifoliolate leaf; SAM, shoot apex (including the apical meristem and immature leaves); T1 to T4, first to fourth trifoliolates (from bottom to top); F, flower buds; P1 to P3, pods at 7, 14 and 21 days after flowering. Data are the means of three biological repeats ± SEM (standard error of the mean). *GLYMA02G10170* (encoding actin in soybean) was used as the internal control.

As noted above, among soybean homologs of FLD, GmFLD and GmLDL2 are more conserved in domain type and organization pattern than LDL1A and LDL1B. This suggests that the functions of GmFLD and GmLDL2 may be conserved in soybean. We thus examined whether *GmFLD* and *GmLDL2* expressions in soybean have similar patterns with that of the Arabidopsis *FLD* and *LDL2*. Figure 3 shows that the transcripts of both genes could be detected in all tissues tested, including roots, hypocotyls and epicotyls, cotyledons, leaves, young pods, and flowers. This indicates that both genes are widely expressed in soybean. However, the transcript levels vary among different organs. The transcript abundance of both *GmFLD* and *GmLDL2* was high in cotyledons, roots and pods, moderate in seedlings, hypocotyls and epicotyls, and flowers, and very low in true leaves, including unifoliate and trifoliate leaves (Figure 3). Interestingly, levels of both *GmFLD* and *GmLDL2* transcripts were also very low in the shoot apex (Figure 3), which is very different from previous reports showing that the Arabidopsis *FLD* and *LDL2* are preferentially expressed in shoot apex [18,19].

### Both GmFLD and GmLDL2 proteins are localized in nuclei

**Figure 4:**
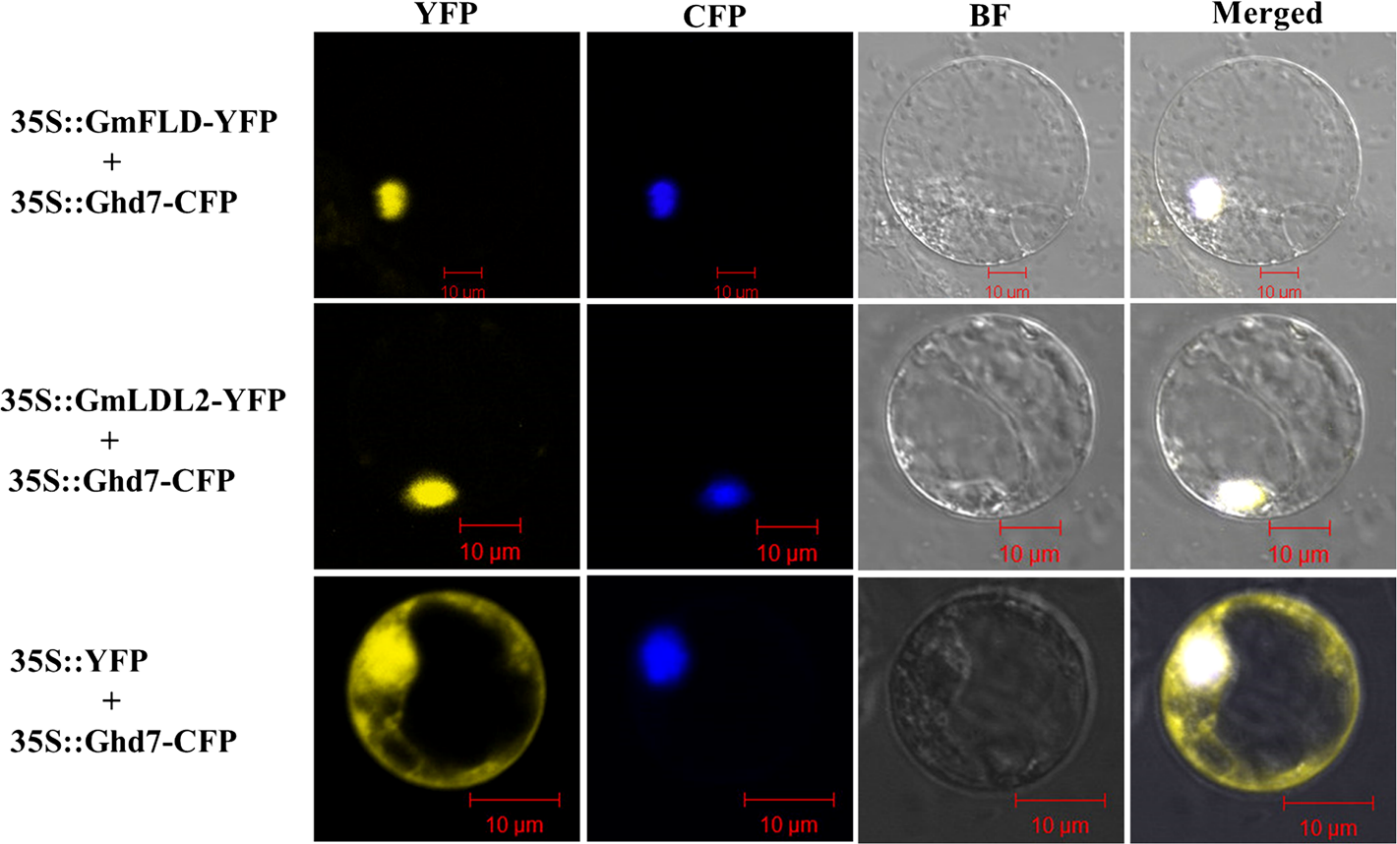
**Subcellular localization of GmFLD and GmLDL2.** Empty vector (*35S::YFP*), constructs *35S::GmFLD-YFP* and *35S::GmLDL2-YFP* were separately co-transferred into rice protoplasts with *35S::Ghd7-CFP* and the fluorescence was examined by using a confocal microscope. Ghd7 was used as a nuclear localization marker.

As putative histone demethylases, GmFLD and GmLDL2 should function in the nucleus. However, a bioinformatics prediction at http://psort.hgc.jp/form.html showed that only GmFLD has putative nuclear localization sites (NLS) and may localize in the nucleus, while GmLDL2 was predicted to localize in the mitochondrial matrix space or cytoplasm. Hence, a transient expression assay was performed to examine the subcellular localization of GmFLD and GmLDL2. The constructs 35S::GmFLD-YFP and 35S::GmLDL2-YFP were used respectively to co-transform rice protoplasts with 35S::Ghd7-CFP, a marker for nuclear localization [36]. Figure 4 shows that yellow fluorescent protein (YFP) signals of both GmFLD and GmLDL2 were clearly overlapped with Cyan Fluorescent Protein (CFP) signals, and no significant fluorescence signals were detected in the cytoplasm. This indicates that both GmFLD and GmLDL2 are localized in the nucleus. This subcellular localization pattern is consistent with the putative functions of GmFLD and GmLDL2 as histone demethylases.

### *GmFLD* but not *GmLDL2* promotes flowering in *A. thaliana*

**Figure 5:**
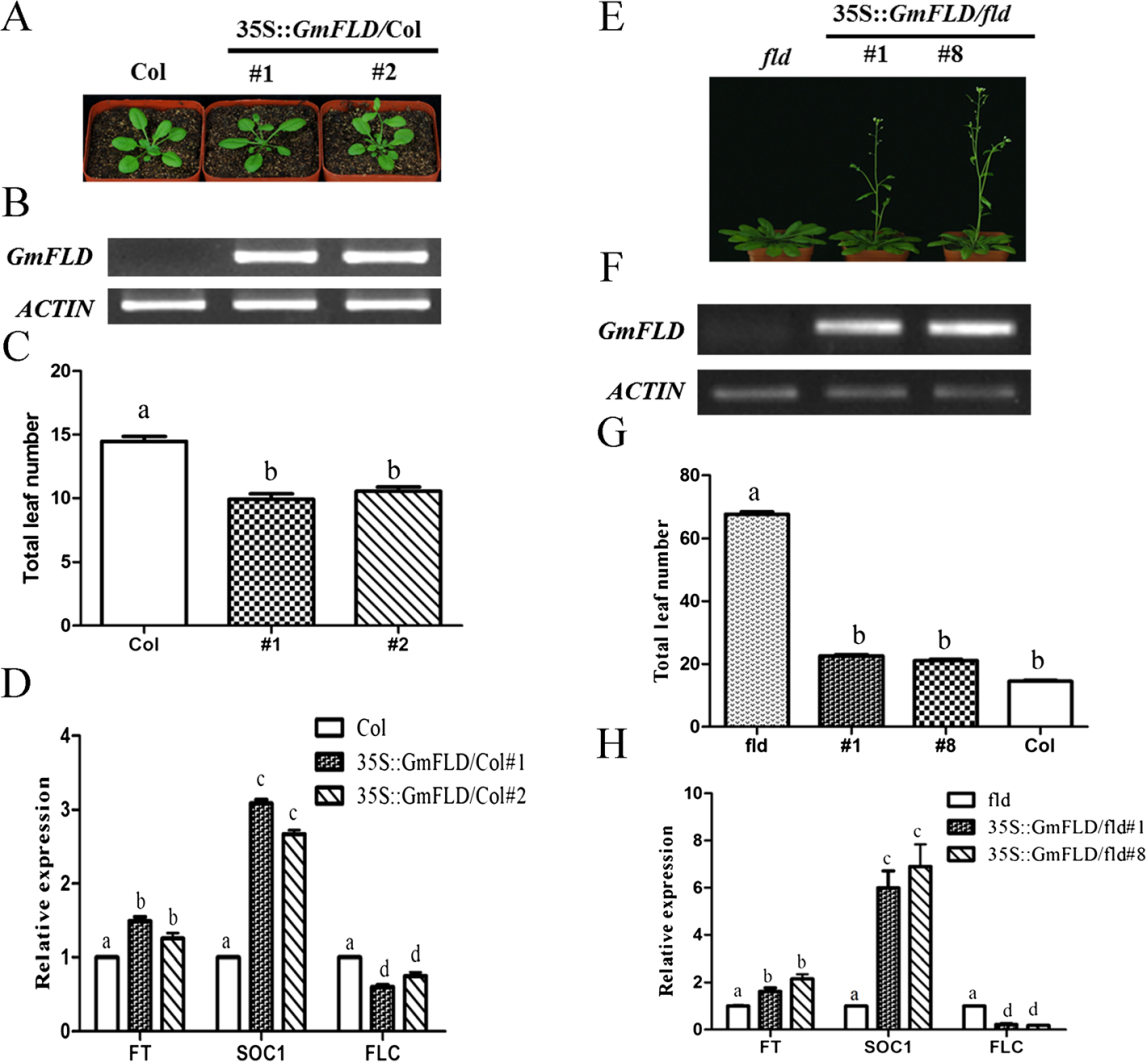
**Heterologous expression of** ***GmFLD*** **in** ***A. thaliana.*** **(A)**, **(E)** Phenotype of transgenic plants. Four **(A)** or eight **(E)** weeks old plants of T3 generation were photographed. **(B)**, **(F)** *GmFLD* expression in transgenic plants detected by RT-PCR. **(C)**, **(G)** Total leaf number of transgenic plants (T3 generation) at bolting. Data are means ± SD. At least twelve plants were counted for each line (n ≥ 12). **(D)**, **(H)** The expression level of *FLC*, *SOC1* and *FT* detected by real-time quantitative PCR. Data are means of three biological repeats ± SEM. *AT3G18780* (*ACT2*) was used as the internal control. Lowercase letters in **(C)**, **(D)**, **(G)** and **(H)** indicate values significantly different at P <0.05. # means independent transgenic lines.

Since *GmFLD* and *GmLDL2* showed different expression patterns from their Arabidopsis counterparts *FLD* and *LDL2* (Figure 3), we examined whether GmFLD and GmLDL2 could function as a flowering time control, similar to the Arabidopsis FLD. Gm*FLD* and Gm*LDL2* CDSs driven by the cauliflower mosaic virus (CaMV) 35S promoter were introduced into *A. thaliana* (Col-0) to assess flowering phenotype of transgenic plants. The transgenic T1 plants expressing *GmFLD* flowered significantly earlier than wild type plants (Table 1), and the early flowering phenotype was also observed in the progenies of the T1 plants (Figure 5A, 5B, 5C). In contrast to *GmFLD*, the transgenic plants overexpressing *GmLDL2* did not show significant changes in flowering time (Table 1).

**Table 1:** **Flowering time of T1 transgenic** ***A. thaliana*** **plants of GmFLD and GmLDL2**

**Genotype**	**Days to visible buds**	**Days to flower opening**	**Rosette leaf number**	**Cauline leaf number**	**N**
Col	28.7 ± 1.8	34.3 ± 1.8	12.3 ± 1.3	3.2 ± 0.7	15
*GmFLD*/Col	24.8 ± 2.1	31.6 ± 2.0	9.4 ± 1.4	2.3 ± 0.5	16
*GmLDL2*/Col	28.1 ± 1.7	34.1 ± 2.0	12.8 ± 0.9	2.8 ± 0.7	16
*fld*	86.5 ± 3.4	95 ± 3.4	59.7 ± 2.5	7.9 ± 1.4	13
*GmFLD*/fld	34.4 ± 8.2	42.8 ± 8.8	10.6 ± 2.5	3.4 ± 1.0	19*

The values are the mean ± SD. N, number of plants scored for phenotype.

*Totally 27 T1 plants were obtained and the flowering time of 19 early-flowering plants was examined. The other eight plants flowered almost as late as the *fld* mutants were not included in this table because no *GmFLD* expression could be detected in these transgenic plants.

### GmFLD complements the late flowering phenotype of *fld* mutant

Since *GmFLD* could promote flowering in the Arabidopsis wild type background, we further checked whether *GmFLD* could rescue the late flowering phenotype of the Arabidopsis *fld* mutant. The *35S::GmFLD* construct was introduced into the *fld* mutant and in the T1 generation, most *GmFLD* transgenic plants flowered as early as the Col wild type plants (19 out of 27 plants flowered early, Table 1). Homozygous single-copy transgenic lines were screened from these early flowering transgenic plants for further analysis. Flowering phenotype scoring showed that the progeny plants consistently flowered much earlier than the *fld* plants, but not as early as the Col wild type plants (Figure 5E, 5G). The *fld* mutants produced approximately 67.6 ± 3.9 leaves (rosette plus cauline leaves) before flowering while transgenic plants produced only 21.9 ± 1.8 leaves, and Col wild type plants produced 14.5 ± 2.3 leaves. The lifecycle of the transgenic plants were also shortened. This was observed by evaluating the time when flower buds became visible or when flowers started to open (Table 1). These results reveal that *GmFLD* could partially complement the phenotype of the *fld* mutant. As expected, *GmFLD* transcript could only be detected in those phenotype-complementary transgenic lines (Figure 5F), whereas in those non-complementary transgenic plants (8 out of 27 T1 plants flowered as late as *fld*), *GmFLD* transcripts could not be detected although the transgene does exist in these transgenic T1 plants (data not shown).

### *GmFLD* promotes flowering in *A. thaliana* through repressing *FLC* transcription

In *A. thaliana*, *FLD* promotes flowering through repressing *FLC* transcription [18,19]. To assess whether GmFLD promotes flowering through the same mechanism, the transcript levels of *FLC* and the floral integrator genes, *FT* and *SOC1*, acting downstream of *FLC* in the transgenic *A. thaliana* (Col-0 or *fld* background) were analyzed. In the transgenic plants in Col background, *FLC* transcript level decreased significantly while *FT* and *SOC1* were up-regulated, the *SOC1* level were especially increased (Figure 5D). Similar trends were observed in the transgenic plants in the *fld* background (Figure 5H). Hence, *GmFLD* promotes flowering in *A. thaliana* through repressing *FLC* transcription as its *A. thaliana* counterpart *FLD* does. Taken the above results together, our experiments demonstrate that *GmFLD* is a functional homolog of the Arabidopsis *FLD*.

### *GmFLD* decreased the levels of histone H3K4me3 and H4 acetylation at the *FLC* locus

**Figure 6:**
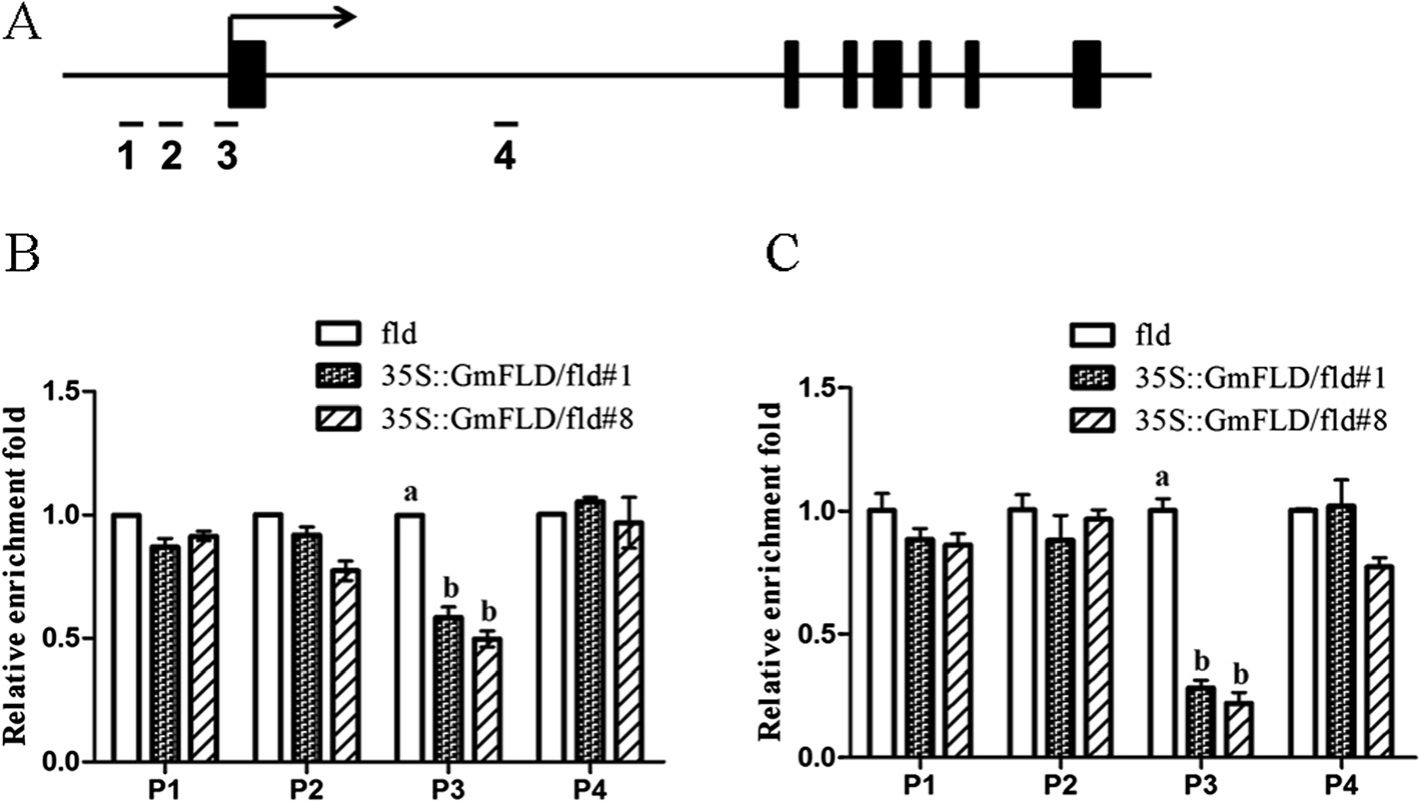
**Chromatin state of** ***FLC*** **in** ***GmFLD*** **-rescued transgenic plants (** ***fld*** **background). (A)** Schematic structure of genomic sequences of *A. thaliana FLC* and the regions examined by ChIP. Arrow: transcription start site and direction; filled boxes: exons; lines: introns. Short lines numbered 1, 2, 3, and 4 depict the positions of PCR amplicons for ChIP. **(B)** Relative levels of trimethyl H3K4 in *FLC* chromatin. **(C)** Relative levels of H4 acetylation in *FLC* chromatin. Data are means of three biological repeats ± SEM using *EIF4A1 (AT3G13920)* as the internal control. Lowercase letters in **(B)** and **(C)** indicate values significantly different at P <0.05. # means independent transgenic lines.

In *A. thaliana*, *FLD* represses *FLC* transcription through affecting the state of H3K4 methylation and H4 acetylation [18,19,37]. So we examined the modification state of H3K4 and H4 in *FLC* chromatin in the transgenic plants that rescued *fld* mutant phenotype. In the *GmFLD* complementing plants, the level of H3K4me3 near the transcriptional start site (P3 region) was significantly decreased, and was about half of that in the *fld* plants. However, we did not find significant changes in the level of H3K4me3 modification in other regions tested (P1, P2, P4) (Figure 6B), which is consistent with previous reports [38,39]. Hence, *GmFLD* could recover at least partially the H3K4 methylation levels of *FLC* chromatin in the *fld* mutant. The acetylation level of H4 in the region around the *FLC* transcription start site was also decreased significantly, whereas no obvious change was found in the P1, P2 and P4 regions tested (Figure 6C). These results suggest that *GmFLD* represses FLC transcription possibly through decreasing the modification levels of H3K4me3 and H4 acetylation in *FLC* chromatin.

## Discussion

Soybean is a typical photoperiod-sensitive crop and photoperiod is an important factor that determines its flowering time. Hence, since the whole genomic sequence of soybean was released [32], the functions of many photoperiod pathway genes, including *GmFT*s, *GmCO*s and *GmGI*s, have been identified and characterized [23,24,26-31,39]. However, little is known about the functions of autonomous pathway genes in soybean, although most of the *A. thaliana* autonomous pathway genes have more than one orthologs in soybean as predicted by bioinformatics analysis [27,33,40]. In this report, the functions of *FLD* homologs in soybean were studied by using bioinformatics, genetic and molecular tools. Our results provide solid evidence to support the function and evolution of autonomous pathway genes in plants.

### *GmFLD* is a functional homolog of *FLD*

In *A. thaliana*, *FLD* plays a major role in promoting flowering, while *LDL1* and *LDL2* play a minor role and act redundantly with *FLD* [18,19]. In palaeopolyploid soybean, both FLD and LDL2 only have a single ortholog, GmFLD and GmLDL2 respectively, and both have functional domains arranged in the same pattern as that in the Arabidopsis FLD and LDL2 (Figure 2). Notably, *GmFLD* could complement the late flowering phenotype of the *A. thaliana fld* mutant plants (Figure 5E, 5G). In the transgenic plants, *FLC* expression is down-regulated and the downstream floral integrator genes *SOC1* and *FT* are up-regulated (Figure 5D, 5H). To our knowledge, no *FLD* homologs from other plants have been experimentally identified and characterized, although the homologs of several other autonomous pathway genes, including *OsFCA* and *OsFVE* of rice, *BvFVE* and *BvFLK* of *Beta vulgaris*, and *ZmLD* of maize [41-44], were studied. Among them, only *BvFLK* and *OsFVE* could complement the late-flowering phenotype of Arabidopsis *flk* or *fve* mutant through *FLC* repression [43,44]. Our ChIP assay further demonstrates that GmFLD is involved in the regulation of the chromatin modification state at *FLC* locus (Figure 6B, 6C). Hence *GmFLD* operated in the transgenic *A. thaliana* in the same manner as the native *FLD* does [18,19]. Although *FLC* orthologs were not identified in leguminous plants previously [45], a recent comparative genomic analysis of soybean flowering genes indicated that soybean has an *FLC* homolog, *GmFLC* (Glyma05g28130) [33]. Whether *GmFLC* is the target of *GmFLD* and (or) other autonomous pathway genes is unknown at present. However from our results, it is reasonable to propose that GmFLD may repress its target gene expression through regulation of the chromatin modification state to control flowering in soybean. Our results suggest that FLD and GmFLD are functionally related regulators of the chromatin modification state and provide the first experimental evidence for evolutionary conservation of FLD function between Arabidopsis and soybean.

### Functional conservation and divergence of *FLD* homologs in soybean

FLD was identified to physically interact with HDA6, a histone deacetylase involved in gene silencing, to function synergistically in chromatin modification [20]. In the complementing transgenic plants, both the H3K4me3 and H4 acetylation levels decreased as compared to those in the *fld* mutant (Figure 6B, 6C). This result suggests that, in addition to its histone demethylase function, GmFLD may also interact with histone deacetylase to affect histone acetylation in chromatin. However, in our preliminary study, interaction between GmFLD and the soybean HDA6 homologs was not detected by yeast two-hybrid analysis (data not shown). Furthermore, the expression pattern of *GmFLD* in soybean is somewhat different from that of *FLD* in *A. thaliana*. The Arabidopsis *FLD* is preferentially expressed in apical meristem regions of roots and shoots, but the transcript level of *GmFLD* is very low in the shoot apex of soybean (Figure 3A). The key function of *FLD* in *A. thaliana* is to promote flowering through repressing the expression of *FLC*, which is epigenetically silenced by vernalization [2,12-15]. Different from *A. thaliana*, soybean does not require vernalization to induce flowering. Therefore, it is conceivable that *GmFLD* probably has additional functions other than flowering control in soybean. Further characterization of the soybean *GmFLD* will be performed in our future work.

In *A. thaliana*, *LDL2* acts redundantly with *FLD* and *LDL1* to repress *FLC* expression, and LDL2 also has overlapping function with *LDL1* to repress sporophytic expression of *FWA* [19]. However, *LDL2* itself plays a minor role in promoting flowering of *A. thaliana*, and loss-of-function *ldl2* mutants do not have significant phenotypic changes [19]. This may explain why heterologous expression of *GmLDL2* in *A. thaliana* did not result in significant flowering phenotype changes. Based on its sequence similarity with LDL2 (Figures 1 and 2), nuclear localization (Figure 4) and expression pattern similar to that of *GmFLD* (Figure 3B), we propose that *GmLDL2* is the functional ortholog of *LDL2* and probably acts redundantly with *GmFLD* to repress the gene expression in soybean. However, its biological roles in soybean still require further investigation. Soybean appears to have two *LDL1* orthologs, *GmLDL1A* and *GmLDL1B*. Interestingly, GmLDL1A and GmLDL1B gained additional functional domains during evolution. The occurrence of TAXi-N and TAXi-C domains in GmLDL1B also suggests functions in pathogen resistance [35].

### Functional divergence of autonomous pathway genes

Autonomous pathway genes were originally identified from a group of *A. thaliana* late flowering mutants and their homologs apparently exist widely in plant kingdom [16,44]. As for *FLD*, two homologs were identified in the genome of *Physcomitrella patens* (Figure 1), a cryptogam without floral transition. This suggests that some *FLD* homologs may play pivotal roles in other developmental processes other than flowering. In cells, autonomous pathway components are involved in chromatin modification and RNA processing, which play important roles in multiple physiological processes such as growth and development, response to abiotic stress, etc. [16,17]. Therefore, it is not surprising that some autonomous pathway genes have additional functions in regulating growth and developmental processes other than flowering. For example, double mutant plants, *fpa fld*, *fpa fve*, and *fpa ld* showed pleiotropic effects on growth rate, chlorophyll content, leaf morphology, flower development, and fertility [46]. Furthermore, some experimental evidence show that both *FCA* and *FVE* play a role in thermosensory flowering pathway [47,48], whereas *FY* is involved in the development of seed dormancy and ABA sensitivity in *A. thaliana* [49]. On the other hand, some orthologs of the *A. thaliana* autonomous pathway genes from other species appear to have diversified in function and/or acting mechanism. Rice *FCA* homolog *OsFCA* could partially rescue the late flowering phenotype of the Arabidopsis *fca* mutant, but through the activation of *SOC1* rather than *FLC* down-regulation. The *OsFCA* also does not have a negative feedback to regulate the Os*FCA* mRNA level as the Arabidopsis *FCA* does [42]. In addition, OsFCA has interaction partners in rice, including OsSF1, OsFIK1 and OsMADS8 [50] that were not identified in Arabidopsis. BvFVE1 of sugarbeet showed 72% amino acid identity to FVE, but could not complement the phenotype of the Arabidopsis *fve* mutant [44], whereas maize *ZmLD* not only failed to complement the *ld* phenotype, but resulted in other developmental defects in *A. thaliana* [41]. Taken the above together, the biological functions of autonomous pathway genes are complex and it is of great interest to probe the biological functions of autonomous pathway components in other plants in addition to *A. thaliana.* Our present data provide the first evidence for evolutionary conservation of the components in the autonomous pathway of flowering in soybean.

## Conclusion

In soybean, FLD has four homologs, GmFLD, GmLDL2, GmLDL1A, and GmLDL1B. GmFLD is a functional ortholog of the Arabidopsis FLD and may play an important role in regulation of the chromatin modifying state in soybean. GmLDL2 is a functional ortholog of LDL2 and may function redundantly with GmFLD in soybean.

## Methods

### Bioinformatics analyses

The *A. thaliana* protein sequences of FLD, LDL1, LDL2, and LDL3 were downloaded from The *A. thaliana* Information Resource [51]. The FLD protein sequence was used to search NCBI [52] and JGI phytozome soybean databases [53] using the blastp algorithm. At this round of search, four FLD homologs (E value = 0.0) were obtained: LOC100786453 (Glyma02g18160), LOC100809901 (Glyma06g38600), LOC100783933 (Glyma07g09980), and LOC100810687 (glyma09g31770). To help infer orthology by bidirectional best hit (BBH) analysis [54], the soybean protein sequences retrieved through the above analysis were used as queries to blast the TAIR10 proteins dataset [51]. For phylogenetic analysis, putative FLD homologs in other plants were identified and retrieved from NCBI database as described above. The rooted phylogenetic tree was constructed by Mega 5.0 [55] and the conserved protein domains were identified using PFAM 27.0 [56]. The subcellular localization of GmFLD and GmLDL2 was predicted through online analysis [57].

### Plant materials and growth conditions

The soybean cultivar Zhongdou32 (*Glycine max L. Merr*.) was used in this study. The soybean plants were grown in pots with soil/vermiculite mixture (V/V = 1:1) in a growth chamber in short-day conditions (8 h light and 16 h dark) at 24-26°C. All *A. thaliana* materials*,* including Col, *fld-1* mutant [18] and other transgenic lines, were grown in long days conditions (16 h light/8 h dark) at 22°C in soil/vermiculite mixture or ½ MS agar plates according to experimental requirement. All *A. thaliana* seeds were stratified for 2 days at 4°C before being moved into the growth chamber.

### Expression pattern analysis of soybean *FLD* homologs

For RNA extraction, plant samples were collected as follows: seedling samples were harvested at the stage when the cotyledons expanded fully. At the unifoliolate stage (when unifoliolates expand fully), hypocotyl, epicotyl, cotyledon and shoot apex (including the apical meristem and leaf primordia) materials were collected. At the flowering stage (when flowers start to open), the flowers, the 1^st^, 2^nd^, 3^rd^ and 4^th^ trifoliolates (from bottom to top) were harvested. The root and unifoliolate samples were collected separately at both the unifoliotate and flowering stages. The pods were sampled separately at 7, 14 and 21 days after flowering. All samples were frozen in liquid nitrogen and stored at −80°C until use.

Total RNA was extracted from seedlings, roots, hypocotyls, epicotyls, leaves, flowers, and pods using Trizol reagent (Invitrogen, USA) according to the manufacturer’s instructions. cDNA was synthesized by using Prime Script™ RT regent Kit with gDNA Eraser (Takara, Japan). The real-time quantitative PCR was performed on a C1000 Touch TM Thermal cycler with SYBR Premix Dimer Eraser™ (Takara, Japan). Each assay was quantified in triplicate and normalized using the actin-encoding gene, glyma02g10170, as an internal control. All experiments had three biological replicates. The primers were listed in Additional file 2 (the same for other primers described below).

### Subcellular localization assay

Full-length CDSs of *GmFLD* and *GmLDL2* were amplified by RT-PCR from the seedling RNA sample and inserted into the vector pM999 at SacI and NcoI restriction sites to generate the transient expression constructs 35S::GmFLD-YFP and 35S::GmLDL2-YFP. The constructs were sequenced and introduced into rice protoplasts according to the method described by Bart et al. [58] and Wang et al. [59]. In brief, about 30 μg endotoxin-free construct DNA was used to transform rice protoplasts. The construct 35S::Ghd7-CFP was used as the nuclear localization marker while the 35S::YFP was used as an empty control [36]. The transformed cells were observed and imaged under the confocal laser scanning microscope (Zeiss LSM dater server). For each subcellular location analysis, at least three biological replicates were performed and at least 10 cells were examined in each sample.

### Heterologous expression of soybean *FLD* homologs in *A. thaliana*

The full length CDSs of *GmFLD* and *GmLDL2* were amplified from the soybean cDNA and cloned into the vector pBI121 at the Xbal and SacI restriction sites, downstream of 35S promoter of cauliflower mosaic virus, to produce the over-expression binary vectors pBI121-GmFLD and pBI121-GmLDL2. After being sequenced, the constructs were introduced into the *Agrobacterium tumefaciens* strain GV3101 for *A. thaliana* transformation. The *A. thaliana* plants (Col wild type or *fld* mutant) were transformed by the floral dip method [60]. The harvested seeds (T1 generation) were selected on 1/2 MS agar media containing 50 mg/L kanamycin. The positive T1 seedlings were transferred to soil/vermiculite mixture to grow for phenotype assay and collection of T2 seeds. The T2 seeds were sowed on kanamycin plates to examine the copy number of the transgene. Only the single-copy transgenic lines were further propagated for producing T3 transgenic homozygous seeds for further experiments. The flowering time was assessed by numbers of rosette and cauline leaves.

The expression of *GmFLD* in transgenic *A. thaliana* was determined by semi quantitative RT-PCR. The expression of *FLC*, *SOC1*, and *FT* in transgenic *A. thaliana* was examined by using real-time quantitative RT-PCR. The RNA was extracted from the seedlings (ten days old) growing on 1/2 MS agar plates according to the method described above. Each assay was quantified in triplicate and normalized using *ACT2* (AT3g18780) as an internal control. All experiments had three biological replicates.

### Chromatin immuno-precipitation (ChIP) analysis

ChIP analysis was performed according to the protocols described previously by Jiang et al. [19]. The leaves from four-week-old *A. thaliana* plants were harvested for experiment. Anti-trimethyl-histone H3K4 and anti-acetyl-histone H4K5K8K12K16 were purchased from Millipore Corporation. The amounts of immuno-precipitated genomic DNA were determined by real-time quantitative PCR. Each assay was quantified in triplicate and normalized using *EIF4A1* (AT3g13920) as an internal control. All experiments had three biological replicates.

## Additional files

Additional file 1:
**Schemas of** ***FLD*** **homologs.** Green box: exon; line: intron. Additional file 2:
**Primers information.**
