# Supplementary figures and images for: GmFLD, a soybean homolog of the autonomous pathway gene FLOWERING LOCUS D, promotes flowering in Arabidopsis thaliana
Source: BMC Plant Biol. 2014 Oct 7;14:263. doi: 10.1186/s12870-014-0263-x (PMC4190295; doi:10.1186/s12870-014-0263-x)

## Slide 1
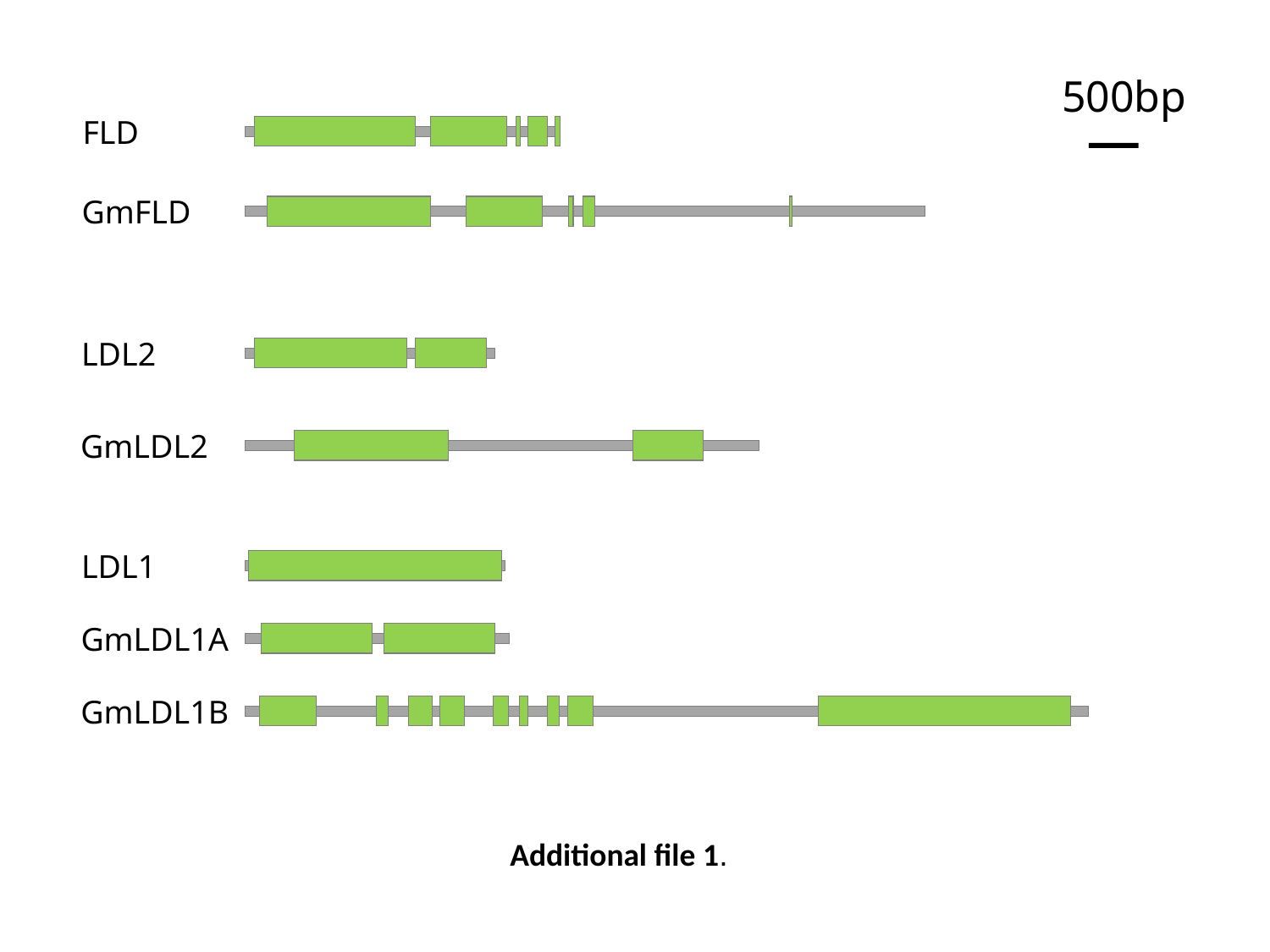

500bp
FLD
GmFLD
LDL2
GmLDL2
LDL1
GmLDL1A
GmLDL1B
Additional file 1.

Supplement: Additional file 1: — Schemas of FLD homologs. Green box: exon; line: intron. [file 12870_2014_263_MOESM1_ESM.ppt]
