# Supplementary material for: GmFLD, a soybean homolog of the autonomous pathway gene FLOWERING LOCUS D, promotes flowering in Arabidopsis thaliana
Source: BMC Plant Biol. 2014 Oct 7;14:263. doi: 10.1186/s12870-014-0263-x (PMC4190295; doi:10.1186/s12870-014-0263-x)
Supplement: Additional file 2: — Primers information. [file 12870_2014_263_MOESM2_ESM.doc]

**Additional file 2 Primers information**

**Primers for transient expression constructs:**

GmFLD-F1: ATCGAGCTCATGGATCCCCCACTCCAATT (SacI)

GmFLD-R1: CATGCCATGGCTGAATTTGTCAAGTCATTTTGATTG (NcoI)

GmLDL2-F1: ATCGAGCTCATGGAAACCCCGTCTTCAA (SacI)

GmLDL2-R1: CATGCCATGGCACAAGTAATTACACGGTTCCTCC (NcoI)

**Primers for ectopic expression in *A. thaliana*:**

GmFLD-F2: ATCTCTAGAATGGATCCCCCACTCCAATT (XbaI)

GmFLD-R2: ATCGAGCTC TTATGAATTTGTCAAGTCATTTTGATT (SacI)

GmLDL2-F2: ATCTCTAGAATGGAAACCCCGTCTTCAA (XbaI)

GmLDL2-R2: ATCGAGCTC TTAACAAGTAATTACACGGTTCCTC (SacI)

Q**-PCR Primers for transcripts level checking:**

qGmactin–F (Glyma02g10170): CTTCTCTCGCTCTCTGCCTTCCAT

qGmactin-R (Glyma02g10170): CACCAGAATCCAACACAATACCAGTTGTA

qGmFLD-F: GGATGGCCATCGTTCACC

qGmFLD-R: CCTTGCCCCCACAGATGTT

qGmLDL2-F: GCACAAGCCTTTGAATCCACAG

qGmLDL2-R: CGGCACCAGATGAATTAACGC

qActin-F: TGTCGCCATCCAAGCTGTTCTCT

qActin-R: CCATCGGGTAATTCATAGTTCTTCTCG

qFT-F: CAACCCTCACCTCCGAGAAT

qFT-R: CGCGAGTGTTGAAGTTCTGG

qSOC1-F: ATTCGCCAGCTCCAATATGC

qSOC1-R: CTGTTGCAGCTCCTCGATTG

qFLC-F: AACACCTTGAGACTGCCCTC

qFLC-R: CACCGGAAGATTGTCGGAGA

**Q-PCR primers for ChIP analysis:**

eIF4A-F: TGACCACACAGTCTCTGCAACTC

eIF4A-R: ATAACCAGGGAGACTTGTTGGAC

FLC-CHIP-qmPCR-1F: GCTGATACAAGCATTTCACCAAA

FLC-CHIP-qmPCR-1R: CTTAAATGTCCACACATATGGCAAT

FLC-CHIP-qmPCR-2F: TGTAGGCACGACTTTGGTAACAC

FLC-CHIP-qmPCR-2R: GCAGAAAGAACCTCCACTCTACATC

FLC-CHIP-qmPCR-3F: CGACTTGAACCCAAACCTGA

FLC-CHIP-qmPCR-3R: GGATGCGTCACAGAGAACAGA

FLC-CHIP-qmPCR-4F: GCCTTGGAATTGTCGAGACAC

FLC-CHIP-qmPCR-4R: TAACTGAGTAACTAAGGGTTCCACG
